# Loss of imprinting of insulin-like growth factor 2 is associated with increased risk of lymph node metastasis and gastric corpus cancer

**DOI:** 10.1186/1756-9966-28-125

**Published:** 2009-09-09

**Authors:** Yang Lu, Ping Lu, Zhi Zhu, Huimian Xu, Xike Zhu

**Affiliations:** 1 Research Center for Medicine, China Medical University the Shengjing Hopital, No. 36, Sanhao ST., Heping District, Shenyang, 110004, PR China; 2 Department of Surgical Oncology, China Medial University the First Affiliated Hospital, No. 155, Nanjingbei ST, Heping District, Shenyang, 110001, PR China

## Abstract

**Background:**

The aim of this study was to determine the clinicopathological features of gastric cancers with loss of imprinting (LOI) of LIT1. Insulin-like growth factor 2 (IGF2) and H19 in Chinese patients.

**Methods:**

DNA and RNA from tumours were amplified and then digested with RsaI, ApaI and HinfI, and RsaI respectively to determine the LOI status. The demographic and clinicopathological characteristics in LOI positive and LOI negative patients were compared and tested with Statistical analysis.

**Results:**

Of the 89 patients enrolled for analysis, 22, 40 and 35 were heterozygous and thus informative for LIT1, IGF2 and H19 LOI analyses respectively. The positive rate of LIT1, IGF2 and H19 LOI of gastric cancer tissues were 54.6% (12/22), 45% (18/40) and 8.6% (3/32) in Chinese patients. Gastric corpus cancer (8/10, 80%) were more likely to have LOI of IGF2 in tumours than antrum cancers (10/30, 33.3%){odds ratio (OR) = 8, 95% confidence intervals (CI) = 1.425-44.920, p = 0.018)}. LOI of IGF2 in tumours was also associated with the lymph node metastasis (LNM) (OR = 4.5, 95% CI = 1.084-18.689, p = 0.038).

**Conclusion:**

IGF2 LOI is present in high frequency in Chinese gastric cancer patients, especially those with gastric corpus cancer.

## Background

Genomic imprinting is an epigenetic modification that leads to the preferential or exclusive expression of a gene from one of the two parental alleles in somatic cells [1]. Abnormal imprinting involved in a number of human diseases, particularly, LOI is one of the most frequent genetic alterations in cancers [2]. LOI can result in either activation or silencing of the normally silent or expressed allele of a growth promoting gene or a growth inhibitory gene, respectively. Research suggests that disruption of imprinting mechanisms may play a critical role in the development of cancer [3]. The cluster of imprinted genes on human chromosome 11p15.5 comprises two imprinted domains: the IGF2-H19 domain and the KCNQ1 domain [4]. H19 and IGF2 genes are imprinted genes and expressed differently depending on whether they are carried by a chromosome of maternal or paternal origin [5]; normally IGF2 expression is coordinately regulated with the maternally expressed H19 gene that produces a noncoding RNA. But in bladder cancer, paternal hypomethylation leads to biallelic H19 expression [6], whereas in Wilms'tumor, maternal hypermethylation and biallelic IGF2 expression are common [7,8]. The level of H19 RNAs in Wilms'tumor is also found to inversely correlate with levels of IGF2 mRNA [9], H19 RNAs were found in polysomes, indicative of H19 translation and/or potential transregulation of IGF2 translation. The upstream promoter region of H19 has the imprinting-control region (ICR) or CTCF binding sites, where the methylation status of this region is critical to the regulation of imprinting of the H19/IGF2 locus located in chromosome 11p15 [10]. LOI of IGF2 is coupled to abnormal H19 methylation in the Wilms tumor case [11]. There may also be an independent mechanism for regulating IGF2 in Beckwith-Wiedemann syndrome (BWS) patients [12]. IGF2 encodes a potent mitogenic growth factor that is active in early development and plays an important role in embryonic and fetal growth [13]. Increased expression of IGF2 is a common feature of both pediatric and adult malignancies since IGF2 binds to the IGF1 receptor to initiate intracellular signaling cascades that lead to cell proliferation [14]. IGF2 stimulates cell proliferation and development in normal human growth. Study showed the overexpressed IGF2 gene is a growth factor for tumors mediated through both the paracrine and autocrine pathways in human cancers. The IGF2 gene may thus play an important role in lymph vessel permeation especially in expanding-type gastric cancers [15]. LOI of IGF2 gene is an important cause of biallelic expression of IGF2 and has been reported in many different types of tumors including osteosarcoma [16], lung adenocarcinomas [17], head and neck squamous cell adenocarcinomas [18], Wilms'tumor [7], prostate cancer [19], and colorectal carcinomas [20]. Studying mice with Apc-Min/+ model of human familial adenomatouspolyposis showed excessive expression of IGF2 resulted increase in the number and the diameter of colon adenoma and increased susceptibility to colon carcinoma [21]. Moreover LOI of IGF2 might provide a marker for identifying an important subset of the population with cancer or at risk of developing cancer [22]. Normally the KvDMR1 in intron 10 of KCNQ1 unmethylated paternally promote LIT1/KCNQ1OT1 expressed paternally antisense RNA [23]. The human LIT1 transcription unit lies within the 11p15.5 imprinted gene cluster and functions as non-coding RNA [24]. Aberrations of LIT1 expression, such as those caused by LOI, involving aberrant hypomethylation and activation of the normally silent maternal allele and LOI IGF2 have been observed in Beckwith-Wiedemann syndrome (BWS) and colorectal cancer [23,25]. In addition, loss of maternal-specific methylation at the LIT1 locus in BWS and several cancers correlates with abnormal imprinting status of CDKN1C [26]. Soejima et al. have recently shown that loss of CpG and histone H3 methylation at a differentially methylated region (DMR)-LIT1 leads to a reduction of CDKN1C expression in esophageal cancer [27]. LOI of IGF2 in gastric tumour tissue except from Taiwan in Chinese and in Japanese patients [15,28] and the clinicopathological features of gastric cancers with LOI of has been reported rarely. In this study LIT1, IGF2 and H19 imprinting status in gastric cancer (GC) which is the second most common cause of cancer-related death in the world was investigated and whether LOI of LIT1, IGF2 and H19 are associated with clinicopathological features was evaluated. The study showed LOI of IGF2 is associated with gastric corpus and LNM in gastric cancer tissues, suggesting that IGF2 plays an important role in gastric carcinogenesis.

## Methods

### Tissues and information collection

The panel of gastric tissues consisted of paired fresh normal adjacent-tumorous and tumorous specimens from 89 GC patients during surgery before any other treatment in the Department of oncology, China Medical University affiliated the first Hospital from March 2007 to February 2008. Written informed consents were obtained from all patients. Demographic and clinicopathologic information were collected from each patient. Tumour location was classified as gastric antrum, gastric corpus, gastric cardia cancer. The tissues were frozen immediately in an -80°C freezer until use.

### Nucleic acid preparation

After homogenizing the frozen tissues, genomic DNA was extracted using standard procedures with phenol/chloroform and precipitated with ethanol. RNA was extracted from grounded tissues using guanidinium isothiocyanate-phenol solution (RNAzol B, Biotecx Laboratories. Inc., Houston, TX, USA) following the manufacturer's instructions. RNA was treated with Rnase-free DNaseI to eliminate DNA contamination (BRL, Baltimore, MD, USA) and stored at -80°C until use.

### Analysis of informative LIT1, IGF2 and H19 cases

Firstly, analyses were performed from DNA of normal tissues to determine informative cases. Heterozygosity in the LIT1, IGF2 and H19 gene was determined by the presence or absence of RsaI, ApaI and HinfI, and RsaI sites respectively. Informative genomic heterozygotes for the LIT1, IGF2 and H19 were studied as follows. The polymorphic region of LIT1, IGF2 and H19 were amplified with the primers [25,18] The PCR reaction was conducted in 1 × PCR buffer with 1 μm primers, 200 μm dNTP, 2.5 units Taq DNA polymerase (Perkin-Elmer, Foster City, CA, USA) and 200 ng genomic DNA. Conditions for amplification were 94°C for 2 min followed by 30 cycles at 94°C for 30 sec, 54 c, 56°C and 58°C (for the LIT1, IGF2 and H19 respectively) for 1 min, and 72°C for 1 min. A final step was 72°C for 5 min. The PCR products were subject to RsaI, ApaI and HinfI and RsaI (New England Biolabs, Beverly, Mass, USA) enzyme digestion at 37°C overnight, run through 12% acrylamide gel and stained with ethidium bromide respectively. The expected size of the PCR fragment of the LIT1 gene is 410 bp. Informative heterozygous cases exhibit three bands of 188, 222 and 410 bp. For IGF2 Primers P1 and P3 were also used to get a 1.4 kb DNA fragment that was used as a size control for the RT-PCR product. PCR conditions were the same except for a 1.5-min annealing step at 60°C C with primers P1 and P3. The PCR products for IGF2 resulted in a 292 bp band with primers P2 and P3. Informative cases are those in which one allele had an ApaI restriction site (256 bp) and the other had an HinfI restriction site (231 bp). The resultant 655 bp PCR product of H19 yielded additional 487 bp and 168 bp bands in heterozygotes.

### Analysis of LOI of LIT1, IGF2 and H19

RT-PCR at LIT1, IGF2 and H19 were further analysed for possible allele-specific expression. One microgram total RNA from heterozygous normal and tumor samples was reverse transcribed for the first strand cDNA using the AMV-RT-PCR system (Sangon, Shanghai, China) in a 20 μl reaction. This reaction mixture was added to 80 μl of 100 μM dNTP and 2 mM MgCl2, 10% glycerol and 2.5 units Taq polymerase in 1 × PCR buffer. Amplification conditions were carried out as described above. For negative PCR controls, the same primers and reaction conditions with RNA, minus the reverse transcription step were performed. After RsaI digestion of RT-PCR products, informative cases of LIT1 with LOI show biallelic expression of both the 222 and 410 bp, while without LOI, showing 222 or 410 band. For IGF2, the RT-PCR product was analysed on 1.5% agarose gel to verify the 1.12 kb bands, which were smaller than those observed in DNA analysis (1.4 kb) with the inclusion of 280 bp intron. Nested PCR wascontinued with the primer P2 as P3 from this 1.12-kb RT-PCR product, resulting in a 292-bp band. After digesting the 292-bp cDNA product from the above RT-PCR reaction with ApaI and HinfI, the presence of 256-bp and 231-bp fragments in a tumor sample indicated biallelic expression. The presence of either the 256 bp or 231 bp band was considered as retention of imprinting. RT-PCR products of H19 resulted in an obvious 575 bp band from cDNA compared to the control of 655 bp fragment from genomic DNA which includes 80 bp intron. Constitutive imprinting yielded either a single 575-bp band or 407- and 168-bp bands, LOI resulting in 575-bp, 407- and 168-bp fragments after RsaI digestion. The threshold for scoring LOI was defined as a ratio of less than 3-fold difference in expression between two alleles [29].

### Statistical analysis

The prevalence of LOI in patients with gastric cancer was described as a proportion. The demographic and clinicopathological characteristics in LOI positive and LOI negative patients were compared and tested using the Chi-Square test. Logistic regression analyses were used to compute the odds ratios (ORs) and 95% confidence interval (CI). Independent sample t-test was used to compare the mean age differences between LOI-positive and -negative patients. All statistical analyses were performed with statistical software with SPSS version 13.0 for windows (SPSS, Inc., Chicago IL). All p-values were two-tailed with 0.05 as statistical significance.

## Results

### Loss of imprinting at LIT1, IGF2 and H19 in gastric cancer tissues

**Figure 1:**
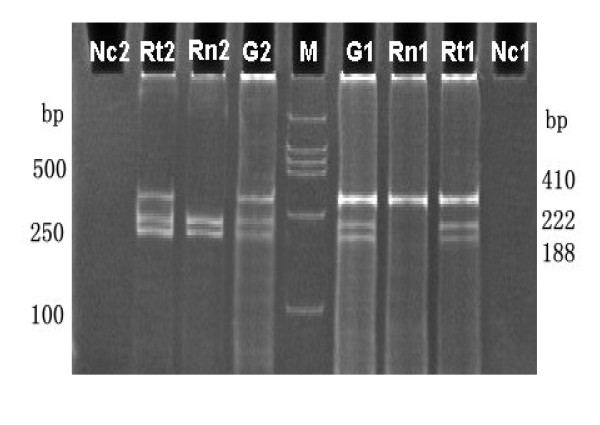
**Imprinting analysis of LIT1 in gastric cancer**. RsaI digestion of a 410 bp DNA PCR product (G1, G2) yielded bands of 222 and 188 bp indicating heterozygous specimens. RsaI digestion of RT-PCR amplification (Rn1, Rn2) showed only one allele expression in both normal tissues indicating maintenance of constitutional imprinting. Rt1, Rt2 displayed three bands in tumor specimens indicating loss of imprinting in contrast to their matching normal tissues (Rn1, Rn2). M, marker DL2000. Nc1, Nc2 represented RT-PCR without reverse transcriptase.

**Figure 2:**
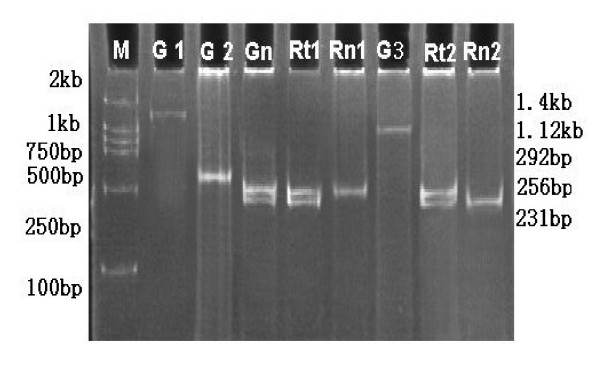
**Imprinting analysis of IGF2 in gastric cancer**. DNA (G1) and RT-PCR (G3) amplification using primers P1 and P3 and DNA amplification by PCR with primers P2 and P3 (G2) represented 1.4 kb, 1.12 kb and 292 bp respectively (see details in methods section). G1, G2 and G3 are PCR products of the same normal tissue. ApaI- and HinfI-digested normal tissue DNA PCR (Gn) from primers P2 and P3 displayed two bands of 256 and 231 bp indicating heterozygosity. The digested nested PCR product from primers P2 and P3 using the 1.12 kb RT-PCR product as a template showed monoallelic expression of IGF2 in normal (Rn1, Rn2) and biallelic expression in tumor (Rt1, Rt2) tissues.

**Figure 3:**
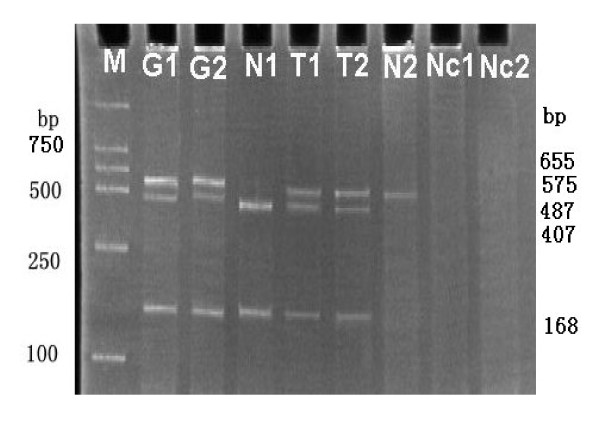
**Imprinting analysis of H19 in gastric cancer**. H19 heterozygosity showed 655 bp DNA PCR product yielded bands of 487 and 168 bp by RsaI digestion (G1, G2). Normal tissues (N1, N2) showed only one allele expression indicating maintenance of normal imprinting (displayed 407 and 168 bp, 575 bp respectively by RsaI digestion RT-PCR products). T1, T2 displayed both three bands (575, 407 and 168 bp respectively) in tumor tissues indicating loss of imprinting in contrast to their matching normal tissues (N1, N2). M, marker DL2000. Nc1, Nc2 represented RT-PCR without reverse transcriptase.

We examined the status of genomic imprinting of the LIT1, IGF2 and H19 genes in 89 gastric cancers by PCR-restriction fragment length polymorphism (RFLP) analysis (Fig. 1, Fig. 2, Fig. 3). Of the 89 tumours analysed, 22, 40 and 35 cases were heterozygous and thus informative for LIT1, IGF2 and H19 LOI analyses respectively as shown in Table 1. LIT1 LOI was observed in 12 of the 22 (54.6%) informative cases, and its LOI was observed in tumor tissues except only one (4.6%) LIT1 LOI observed in the adjacent normal tissues. IGF2 LOI was observed in 18 of the 40 (45%) informative cases, and all the cases showed LOI in the adjacent normal tissues. In five cases LOI were observed in the normal tissues, but not in the cancer ones. Only one informative case showed LOI for both LOI LIT1 and IGF2. We observed only 3 LOI H19 of the 32 (8.6%) informative tumors cases, and two cases showed LOI in cancer tissues. In one case, LOI was observed in the normal tissue, but not in the cancerous tissue.

**Table 1:** Summary of allele-specific expression in 89 gastric cancers

**Gene**	**Informative(*n*)**	**Imprint**	**LOI**	**Incidence of LOI in tumor**
LIT	22	10	12	12/22 (54.6%)

IGF2	40	22	18	18/40 (45%)

H19	35	32	3	3/32 (8.6%)

### Demographic analysis

The demographic characteristics of patients with or without LOI of LIT1, IGF2 and H19 were shown in Table 2. There were no differences in the mean age, sex ratio, diabetes mellitus(DM), cigarette smoking, alcohol consumption, and family history of GC between the LIT1, IGF2 and H19 LOI(+) versus (-) respectively.

**Table 2:** Demographic data of patients with and without LIT1, AIGF2 and H19 LOI

	**LIT1** **LOI (+)** **N = 12**	**LIT1** **LOI (--)** **N = 10**	**P-value**	**IGF2** **LOI (+)** **N = 18**	**IGF2** **LOI (--)** **N = 22**	**P-value**	**H19** **LOI(+)** **N = 3**	**H19** **LOI (--)** **N = 32**	**P-value**
Gender			1.000			.749			.708

Male	9 (75%)	8 (80%)		14 (77.8%)	15 (68.2%)		3 (100%)	23 (71.9%)	

Female	3 (25%)	2 (20%)		4 (22.2%)	7 (31.8%)		0	9 (28.1%)	

Mean age, yr (SD)	64.5 (12.4)	61.3 (13.9)	0.574	58.6 (12.4)	62.7 (14.0)	.344	68.3 (13.4)	59.7 (13.8)	.306

Family history of GC	4 (33.3%)	0	0.096	0	4 (18.2%)	.168	0	4 (12.5%)	1.000

DM	0	1 (10%)	0.455	2 (11.1%)	0	.196	0	2 (6.25%)	1.000

Cigarette smoking	10 (83.3%)	7 (70%)	0.816	13 (72.2%)	13 (59.1%)	.386	2 (66.7%)	22 (68.8%)	1.000

Alcohol consumption(>10 g/day)	4 (33.3%)	3 (30%)	1.000	6 (33.3%)	5 (22.7%)	.695	2 (66.7%)	9 (28.1%)	.227

LOI: loss of imprinting; SD: standard deviation; GC: gastric cancer; DM: diabetes mellitus

### Clinicopathological features according to LOI LIT1, IGF2 and H19 status and factors associated with positive LOI IGF2

Of the 40 informative IGF2 tumour samples, 30 tumours were located at the antrum and 10 tumours were located at the gastric corpus. Gastric corpus cancer (8/10, 80%) were more likely to have LOI of IGF2 in tumours than antrum cancers (10/30, 33.3%) (p = 0.028) and the positive rate of LOI IGF2 was significantly higher in patients with lymph node metastasis than in those without (69.2% versus 33.3%, p = 0.033) as shown in Table 3. There were no differences in the histological differentiation,, hepatic and peritoneal metastasis, lymphatic or venous invasion, tumour size, stage, Borrmann type and TNM between the LIT1, IGF2 and H19 LOI(+) versus (-) respectively. And there were no differences in the tumor location and lymph node metastasis between the LIT1 and H19 LOI (+) versus(-) respectively. The LOI positive rate of the LIT1, IGF2 and H19 was higher in patients with advanced tumour stage than with early stage, but the difference was not statistically significant (p = 1.000).

**Table 3:** Association of clinicopathological features with LIT1, AIGF2 and H19 LOI

	**LIT1** **LOI (+)** **N = 12**	**LIT1** **LOI (--)** **N = 10**	**P-value**	**IGF2** **LOI (+)** **N = 18**	**IGF2** **LOI (--)** **N = 22**	**P-value**	**H19** **LOI(+)** **N = 3**	**H19** **LOI (--)** **N = 32**	**P-value**
Tumor location			1.000			.028			.633

antrum,	10 (83.3%)	8 (80%)		10 (55.6%)	20 (90.9%)		3 (100%)	22 (68.8%)	

gastric corpus,	2 (16.7%)	2 (10%)		8 (44.4%)	2 (9.1%)		0	10 (31.2%)	

gastric cardia	0	0		0	0		0	0	

Histological differentiation (well, mod/poor, muc)	5/7	4/6	1.000	9/9	10/12	.775	1/2	15/17	1.000

Lymph node metastasis	5 (41.7%)	4 (40%)	1.000	9 (50%)	4 (18.2%)	.033	1 (33.3%)	12 (37.5%)	1.000

Hepatic and peritoneal metastasis	1 (8.3%)	0	1.000	1 (5.6%)	1 (4.6%)	1.000	0	2 (6.25%)	1.000

Lymphatic invasion	4 (33.3%)	1 (10%)	.323	4 (22.2%)	4 (18.2%)	1.000	0	8 (25%)	.789

Venous invasion	1 (8.3%)	0	1.000	1 (5.6%)	1 (4.6%)	1.000	0	2 (6.25%)	1.000

Tumour Size			.746			.332			.423

<2 cm	0	0		3 (16.7%)	6 (27.3%)		0	6 (18.8%)	

2--5 cm	8 (66.7%)	6 (60%)		7 (38.9%)	11 (50%)		1 (33.3%)	16 (50%)	

>5 cm	4 (33.3%)	4 (40%)		8 (44.4%)	5 (22.7%)		2 (66.7%)	10 (31.3%)	

TNM			.369			.525			.208

T+N+M=<3	7 (58.3%)	3 (30%)		8 (44.4%)	12 (54.6%)		0	18 (56.3%)	

T+N+M>=4	5 (41.7%)	7 (70%)		10 (55.6%)	10 (45.4%)		3 (100%)	14 (43.7%)	

Stage			1.000			1.000			1.000

early	1 (8.3%)	0		0	1 (4.6%)		0	1 (3.1%)	

advanced	11 (91.7%)	10(100%)		18 (100%)	21 (95.4%)		3 (100%)	31 (96.9%)	

Borrmann type			.620			.337			.753

I	1 (9.1%)	0		0	2 (9.5%)		0	2 (6.5%)	

II	0	0		0	0		0	0	

III	9 (81.8%)	9 (90%)		16 (88.9%)	18 (85.7%)		3 (100%)	26 (83.9%)	

IV	1 (9.1%)	1(10%)		2 (11.1%)	1 (4.8%)		0	3 (9.7%)	

Tumours with LOI of IGF2 are associated with increased risk (OR = 8, 95%CI = 1.425-44.920, p = 0.018) of the gastric corpus cancer versus those without LOI and the increased risk of the lymph node metastasis (OR = 4.5, 95%CI = 1.084-18.689, p = 0.038) as shown in Table 4.

**Table 4:** Odds ratio for gastric corpus cancer and lymph node metastasis of the LOI IGF-2

**Variable**	**Patients with gastric corpus cancer**	**OR for gastric corpus cancer (95% CI)**
IGF2 LOI(+)	44.4% (8/18)	8 (1.425-44.920, p = .018)

Normal imprinting	9.1% (2/22)	1

	Lymph node metastasis	OR for lymph node metastasis (95% CI)

IGF2 LOI(+)	50% (9/18)	4.5 (1.084-18.689, p = .038)

Normal imprinting	18.2% (4/22)	1

OR: odds ratio; CI: confidence interval; IGF-2: insulin growth factor 2; LOI: loss of imprinting

## Discussion

The cluster of imprinted genes on human chromosome 11p15.5 consists of two domains: IGF2-H19 domain and the KCNQ1 domain [4]. LOI of IGF2 has been observed in 10% of the lymphocytes from normal individuals [30]. In normal human brain, biallelic expression of IGF2 and/or H19 is found despite differential methylation and CTCF binding [31]. In this study, we have shown that LOI of the LIT1, IGF2 and H19 are present in 54.6%, 45% and 8.6% of gastric cancer tissues in Chinese patients respectively. This is the first study to detect on the LOI of LIT1, IGF2 and H19 in gastric cancer in China-Mainland patients and LOI of IGF2 positive correlation with gastric corpus tumour (OR = 8, 95%CI = 1.425-44.920, p = 0.018) and lymph node metastasis (OR = 4.5, 95%CI = 1.084-18.689, p = 0.038).

The frequency of IGF2 LOI (+) gastric cancers (45%, 18/40) is slightly higher than that reported from Taiwan (34.5%, 10/29) [28]. High frequency of IGF2 LOI was observed in tumor and adjacent normal tissues and Igf2 LOI with Apc+/Min mice showed a shift toward less differentiation and an increase in tumor initiation indicating that IGF2 LOI occur at an early stage in cancer development [32]. Although the mechanisms underlying IGF2 LOI in human cancer remains unknown, it is likely to directly or indirectly involve the H19 ICR. We used the allele-specific restriction enzyme digestion technique to identify LOI status, similar to that reported previously [22]. Some papers have explained the mechanisms in the tumourigenesis of gastric carcinogenesis [33]. A variety of epigenetic alterations in human cancers include global DNA hypomethylation, gene hypomethylation and promoter hypermethylation, and IGF2 LOI. The mechanisms for LOI are hypermethylation or hypomethylation of a DMR upstream of the H19 gene, allowing activation of the normally silent maternal allele of IGF2. LOI may precede the development of cancer and may thus serve as a common marker for risk, but also as a model for understanding the developmental mechanism for normal imprinting. Therefore, it is possible that IGF2 LOI play a role in the tumourigenesis through epigenetic modification of DMR. Positive correlations were identified between elevated IGF2 expression and hypermethylation of CTCF binding sites at the H19 proximal imprint center in ovarian cancer [34]. H19 may be a tumor suppressor gene involved in head and neck carcinogenesis [35]. Epigenetic alterations of H19 or LIT1, which encode untranslated RNAs on 11p15, are strongly associated with cancer risk or specific birth defects in BWS [36].

We found that gastric corpus cancer is associated with higher IGF2 positive LOI rate, while Liou et al [37] found that proximal colon cancer is independently associated with higher positive LOI rate, consistent with a recent report from Japan [38]. However, larger population are needed to screen whether IGF2 LOI is involved in which pathways of gastric carcinogenesis. LOI of LIT1 involves aberrant hypomethylation and activation of the normally silent maternal allele. Our data suggest that LIT1 LOI may be associated with gastric cancer tumorigenesis. Histone modifications and DNA methylation are important for the regulation of LIT1 expression to form active or repressive chromatin structure [27]. LIT1 is a non-coding RNA, like Xist, Tsix and Air, LIT1 RNA plays a critical role in the bidirectional spreading of inactive chromatin structures [39], silencing imprinting genes [40] and formating of the imprinting center (IC) to coordinate imprinting in the 11p15.5 region. Timing of LIT1 RNA expression is vital for the proper initiation of imprinting genes [41,42]. Premature termination of the LIT1 transcript leads to LOI in the proximal region indicating full-length Lit1 RNA is necessary for maintaining the imprinting status [43]. Mouse Lit1 RNA plays a critical role in silencing at the IC of the imprinted gene cluster and the transcript length of Lit1 RNA is important for the degree of silencing [44].

And we found patients with LOI of IGF2 in their tumour had higher increased risk of the lymph node metastasis than those without (OR = 4.5, 95%CI = 1.084-18.689, p = 0.038). Recently our study found metastatic lymph node ratio is a useful independent prognostic factor and may obviate possible confounding factors that are related to stage migration, and should be considered as an important component in the lymph node ategory. Lymph node ratio category has advantages in providing a more precise prognostic value than the pN category (5th edition, UICC). We recommend that classification of nodal status be established by a combination of both the metastatic nodes number and ratio, which would be the best category to provide both rational lymph node dissection and the foundation for adjunctive therapy and predict the prognosis [45]. Ohashi et al reported conventional pathological factors, such as tumor size, depth of submucosal invasion, and lymphatic invasion, have a significant influence on lymph node metastasis in submucosal invasive gastric cancer [46]. Li et al showed depth of invasion, lymph node metastasis, hepatic and peritoneal metastasis and surgical curability were significant factors affecting survival of the gastric carcinoma patients [47]. But we failed to find such an association. Liu et al found transversal and skipping metastases of sentinel lymph nodes (SLN) are notable and therefore rational lymphadenectomy should be performed in primary gastric cancer [48]. Some research demonstrated lymph node metastasis were independent prognostic factors in human gastric carcinoma [49]. And high expression of mitotic centromere-associated kinesin (MCAK) and tripartite motif-containing 29 (TRIM29) are predictors for lymph node metastasis [50,51]. It might be more appropriate that identifying patients at high risk of lymph node metastasis who should be offered gastrectomy rather than endoscopic mucosal resection, because patients with lymph node metastasis are more likely to express IGF2 LOI than those without. Our result was consistent with other studies that LOI of IGF2 is also important in the carcinogenesis [15,28].

## Conclusion

In all, high frequency of IGF2 LOI is present in patients with gastric cancer in the northeast of China. The association of IGF2 LOI with lymph node metastasis may contribute to the development and progression of gastric cancer.

## Abbreviations

GC: gastric cancer; LNM: lymph node metastasis; LOI: loss of imprinting; IGF2: Insulin-like growth factor 2; DM: diabetes mellitus; SD: standard deviation; CI: confidence interval; OD: odds ratio; BWS: Beckwith-Wiedemann syndrome; DMR: differentially methylated region; RFLP: restriction fragment length polymorphism; ICR: imprinting-control region.

## Competing interests

This paper has not been published elsewhere in whole or in part. All authors have read and approved the content, and agree to submit for consideration for publication in the journal. 'The authors declare that they have no ethical, financial or legal competing interests in this article.

## Authors' contributions

YL carried out nucleic acid preparation, PCR, RT-PCR and PCR-RFLP analysis, performed the statistical analysis. PL, HX and ZZ participated in tissues, information collection and PCR- RFLP analysis. ZZ, HX and XZ participated in statistical analysis and helped to draft the manuscript. All authors read and approved the final manuscript.
